# Going for the Octopus

**Published:** 1906-06

**Authors:** 


### Going for the Octopus.

The following six paragraphs are from the *Medical Sentinel* for May, from the pen of the indomitable Henry Waldo Coe:

THE AMERICAN MEDICAL ASSOCIATION GANG.—That the American Medical Association is being run in the interest of a band of Chicago manipulators, is more and more being recognized by the profession throughout the land.

A good deal of fault has been expressed here in Oregon against Dr. McCormack. This is a needless and misdirected assault upon a worthy gentleman. McCormack is a good-natured gentleman, one of the best politicians in America, who is employed by the men who control the A. M. A., through the *Journal,* to go about the country, nominally to "organize" the profession, but actually to feel out the pulse in various localities, lay the wires and provide that at each coming session only the ornamental offices shall go to the "unwashed outside."

Oregon, Washington, and Idaho had the largest percentage of members in the A. M. A. as to the entire number of doctors in these States, of any three States in any neighborhood in the United

States. The Oregon physicians had raised nearly $25,000 to entertain the A. M. A., and had tried to do their duty in the great work of the annual session of the greatest scientific body on earth. The meeting came to Portland against the wishes of the *Journal* ring. As soon as the meeting was well over, this ring thought it well to make an attack upon us. It not only sent their bland politician out here to abuse us, but editorially attacked us. They thought that out in this outskirt of the country, they might find supple knees to bend, and if not, "what could little Oregon do about it?" There are States in the East where the percentage of membership in the A. M. A. is not one-fourth that of Oregon. If "Organization" is what was wanted, why not work there?

A Medical Bureaucracy.—Quite a number of people *think* they are in the ring which controls the *A. M. A. Journal.* As a matter of fact, a half-dozen or at most a dozen people are all who know what the *Journal* is doing.

Reports are sent out yearly of what is done with the very large sums of money which pass through the office of the *Journal*—about a quarter of a million dollars—which read as nice as for years have the life insurance reports. Other medical publishers can not exactly see how so much money is being expended.

Because Oregon and the delegates from the Northwest insisted upon Portland being a candidate for the last meeting of the A. M. A., after the ring had determined upon another place for the meeting, and (perish the thought) even secured such meeting, a walking delegate was sent here, nominally to organize, but in fact to see if he could not find something to criticise. He and the *Journal* then made the heartless attack upon Oregon, of which our readers already know. An attack so cowardly as to be almost unthinkable. "Poor little old Oregon," these assailants evidently thought, "these people way out there can do nothing, and we will show how severe we can be. Others, we trust, will appreciate, and that a word to the wise will be sufficient."

They overlook the fact that every great conflagration with plenty of material at hand has yet had to start in a small place.

The editor of the *Medical Sentinel* was and is President of the Portland City and County Medical Society. As such officer he helped arrange an itinerary for Dr. McCormack in Oregon and Idaho, and allotted one of the regular sessions of the Portland society to this gentleman. For many years there has been a divided opinion in the local society as to limited or more general membership, in which the editor in his individual capacity has been steadily working for the fullest possible membership. The leaders' opposition to fuller membership had long ago conceded the point and membership has been growing by leaps and bounds for the past two years. The increase last year was 35 per cent. This year, following the A. M. A. meeting, 50 per cent. We believe that Portland has a larger percentage of membership to the total number of doctors than any other city of its size in America!

Directors Who Do Not Direct.—In the Life Insurance swindle it has been shown that, although the names of men the very highest in the land have been attached to reports and other printed matter as directors and trustees, almost none of these know anything about the internal workings of the organizations which the public had confidingly presumed such directors were assisting to manage. Of necessity, men living in remote places, far removed from Chicago, can not be expected to know all that is going on there, and their acts must be largely perfunctory. We would not assume that such men, high in the profession as some are, who are nominally at the head of the *A. M. A. Journal,* are knowingly doing wrong. Depew, Morgan and other New Yorkers were not charged with knowingly doing wrong. They merely "didn't know." If the people in the home city of a corporation can not keep in touch with an institution, we may expect less responsibility from those a thousand miles away.

The American Medical Association is the greatest scientific body in the world. Its *Journal* should be the greatest medical periodical and above every suspicion of being used for ulterior purposes by a band of self-seekers who may for the time be in control.

The Self-Effacement of Local Medical Journals. — The medical journals of the United States are much to blame, in which the *Medical Sentinel* admits its own part, in building up the medical ring which now, through the *Journal of the American Medical Association,* has placed itself pretty thoroughly over and above the whole profession of America, in that their columns have always been freely open to anything which should advance the standing of the *Journal.* Personal advantage has been entirely put one side, and the medical journals have worked earnestly and continuously to build up the *Journal of the A. M. A.* into the greatest publication of medicine of the land. The fact that the upbuilding of the *A. M. A. Journal* was certain to do damage to other medical periodicals was well understood by every medical publisher, but the owners of medical journals put their own personal interests one side when considering the *Journal of the A. M. A.*, which they felt was to be the general organ of a great profession.

Having built up the *Journal* to its present position, which it could not have attained in any considerable degree without the aid of the general and local medical press of the United States, one of the first things which those who have managed to secure control of the periodical have attempted has been to drive out of business every other periodical in the land. Today, under the present managment of the A. M. A., in the journalistic field, the *Journal* is out with its hand against every man. It is a true Ishmaelite. The *Medical Sentinel*, standing alone in its own limited field, is free to speak of this phase, for it can not to any great extent be injured by the present methods of the *A. M. A. Journal*. But whether injured or not, and doubtless it is to be made a special object of the kind of insiduous warfare the ring so well knows how to carry on, it shall speak for decency and justice.

Is the A. M. A. Ring "Unbustable"? — When the ring has "properly routed" itself, then the ukase goes out that the meeting for the next year is to be held at the place selected. When the officers have been picked out, then the gum shoe travels of the political supes begin. In each State effort has been made to see that no one is elected a delegate who is "wrong." With a little judicious peddling of blue ribbons and minor offices, the thing is ready to go.

But does it always go?

Not always.

We remember the report of the Place of Meeting Committee at the last session. It was to be kept secret until presented. If it should be discussed, things might go wrong. Like a lot of unruly children, the delegates—eighty or one hundred men—were calmly told that they would not be informed as to the proposed place of meeting until the very end of the meeting.

Chairman Harris, of Chicago, with his patent inside report, was finally ready.

With bated breath the anxious delegates listened.

It should have been a moment for humility for them.

But the delegates wanted Portland. The ukase called for another point.

There were other Chicago doctors in the House of Delegates. One of these was Harold N. Moyer. He is not a member of the Chicago ring. He wanted Portland. Of course, our own Northwest delegates were for Portland. Moyer spoke. Mackenzie spoke. Others spoke. A ballot followed.

All but fifteen votes out of eighty-four were against the ring.

Portland won.

This is a fair illustration of what may follow if the delegates take it into their heads to exercise their constitutional rights.

THE HOUSE OF DELEGATES OF THE A. M. A.—Each State medical society meets in its local sovereign capacity and selects one or more members to the national House of Delegates. In America there is no other body where so few men represent so many learned constituents. These one hundred men, picked on account of high standing, personal ability and special fitness for legislative work, bear the responsibility for the welfare of the American medical profession, 100,000 strong. Their relations to the real work of the Association has been permitted by the ring to confine itself principally to such responsibility.

Think of it!

This body of selected men, leaders in their own localities over the land, waiting in their seats or passing time in immaterial discussions and affairs, anxiously looking to the door of the "inner room," from whence from time to time the real orders are to come.

And this condition—a travesty on justice, a mockery upon liberty, a reflection upon the intelligence of the House of Delegates, and a personal insult to every lover of free manhood in that body —is a condition existing in this year of our Lord 1906!

